# To what extent can language teachers enact agency in emergency online teaching?

**DOI:** 10.3389/fpsyg.2022.922918

**Published:** 2022-08-18

**Authors:** Qiaoying Cai, Xinmin Zheng

**Affiliations:** ^1^College of Foreign Languages, Fujian Normal University, Fuzhou, China; ^2^School of Education, Shanghai International Studies University, Shanghai, China

**Keywords:** language teacher, teacher agency, emergency online teaching, enactment, teacher identity

## Introduction

Without any exaggeration, the abrupt explosion of COVID-19 has brought great challenges to our ways of living, education in particular. In order to prevent the spread of COVID-19, many face-to-face social activities were strictly forbidden. Due to this, teachers' habitual teaching was also disrupted severely and quick adaptation to online teaching was required. This was particularly the case for language teachers who may be have been confronted with the severest disruptions (Ruan and Zheng, 2019; Gao, 2020) as language teaching relies heavily on face-to-face interactions with students. Even though the impact of online environments on language teaching has been well-explored by researchers (Gacs et al., 2020; González-Lloret, 2020; Tucker, 2020; Zhang et al., 2021), little is known about how language teachers respond to emergency online teaching, that is, a temporary shift to teaching online caused by sudden and serious events like COVID-19.

With great interest, we read the article entitled Language Teacher Agency in Emergency Online Teaching by Ashton (2022), which examined language teachers' agency in emergency online teaching context. Specifically, it focused on the practical-evaluative agency of language teachers in a temporary shift to online teaching caused by COVID-19. As we see it, the article has revealed some tricky problems exiting in emergency online teaching, and the research findings and interpretations are illuminating and instructive for both practitioners and researchers. Yet, it seems there is still room to dig deeper from more comprehensive and diverse perspectives to better understand the interplay of the recurrence of COVID-19 and language teachers' agency. Therefore, we would like to comment on the article and share our opinions on language teacher agency in emergency online teaching, in the hope of providing constructive suggestions for future research studies and online language teaching practice.

Prior to analyzing the article in detail, it is necessary to elucidate the two concepts mentioned previously, namely the practical-evaluative dimension of agency and emergency online teaching, in order to help better understand and interpret the article.

The practical-evaluative dimension of agency is, in essence, a key concept of the ecological perspective of agency. Generally speaking, an ecological approach at its core is the chordal triad of agency, that is, the practical-evaluative dimension (present), the habitual dimension (past), and the projective dimension (future). To be more specific, teacher agency is finally enacted based on teachers' reflections on past experiences, expectations of future goals and most importantly practical evaluation of the former two aspects to identify appropriate courses of action in the present Emirbayer (1998). Despite the fact that the ecological perspective of agency is widely applied and greatly developed by a number of researchers (e.g., Priestley et al., 2012, 2013, 2015a,b; Biesta et al., 2017), the practical-evaluative dimension of agency has been particularly neglected and under-theorized (Emirbayer, 1998; Hitlin, 2007), which is exactly the focus of the article.

The other concept we will clarify is emergency online teaching. Emergency online is “a temporary shift of instructional delivery to an alternate delivery mode due to crisis circumstances” (Hodges et al., 2020). To avoid confusion, we think it is necessary to distinguish emergency online teaching from general online teaching. General online teaching differs from emergency online teaching as online curriculum design and pedagogical decisions are carefully planned and considered beforehand (Hodges et al., 2020), whilst emergency online teaching means that language teachers are required to move their teaching online abruptly and have little time to adjust to the new teaching context. As mentioned before, our understanding of language teacher agency in emergency online teaching is rather limited, which necessitates the article's investigation of how language teachers enact agency in the context of online teaching during COVID-19.

## The study

As stated in the introduction, the article (Ashton, 2022) made efforts to explore how language teachers' agentic actions worked in emergency online language teaching context. In this section, a summary will be made about the article including its conceptual framework, method of study, and research findings.

The conceptual framework this study employed is the ecological perspective of agency introduced by Emirbayer (1998), echoed by Priestley et al. (2012), broadened by Priestley et al. (2013, 2015a,b), and further developed by Biesta et al. (2017). Drawing on the ecological perspective of agency, this study focused on the practical-evaluative dimension, namely how teacher agency is enacted in the present, attempting to further the study regarding the practical-evaluative dimension which has been particularly neglected and under-theorized (Emirbayer, 1998; Hitlin, 2007).

Qualitative study in nature, the study adopted the case study approach to examine the agency of four female language teachers who were from different countries with different backgrounds and worked in different institutions in New Zealand in emergency online teaching when COVID-19 broke out. Data were collected through multiple sources, i.e., retrospective interviews, reflection notes, informal conversations and emails, with interviews being the primary source of data, and teacher notes, informal conversations and emails being the secondary source of data. Data analysis was conducted in three stages. Firstly, the data were coded as the affordances and constraints in enacting agency; secondly, the analysis was centered around individual critical incidents; and thirdly, the analysis traced through these incidents and how they impact each participant's agency.

The research findings of this article show that due to the COVID-19 lockdown action by their institutions, all the four case study teachers' routines were disrupted by the sudden shift to emergency online teaching, and they experienced the sense of isolation, loneliness and even hopelessness. Within this larger shared critical incident, this study discusses the unique critical incidents that prompted each teacher to reflect on their practice, the agency they enacted and factors influencing the actions taken. With rich evidence this study explores profoundly and thoroughly how each teacher exercised their agency in line with their professional identities and claims that social structural factors feature prominently in teachers' identities and enactment of agency. Furthermore, with the lens of the practical-evaluative dimension of agency, this study elucidates how the four case study teachers used their agency to maximize the benefits, evaluating the “solution” adopted as an appropriate response to multiple concerns and goals. Obviously, this study has important implications for teacher development highlighting the need for professional development programs to better prepare teachers for the diversity of teaching situations which they may encounter, particularly in relation to differences in the role of the teacher, and the power dynamics and relationships between teacher, students, and parents in different teaching modes.

## Discussion

We appreciate that the article has contributed an interesting point of view with convincing empirical evidences for us to understand how language teachers' agency could be specifically enacted and adapted to the changing context. In this section, based on the article, we would make our comments on the selection of the participants, use of instruments and other possible variables concerning emergency online teaching for the purpose of promoting future study.

First, regarding the selection of the participants, the teachers identified in the case study were all experienced language teachers, most of whom had been in the service for more than 10 years. It is clear that experienced teachers are more mature and more capable of making sound decisions when they face new problems and challenges in the teaching context (Zheng and Borg, 2014; Burkhauser, 2017). We think it would be a good idea to include some novice teachers to examine how they cope in this tide of emergency online teaching. For many novice teachers, the first solo effort in emergency online teaching context is a “sink or swim” experience. New in the field and with less experiences, especially when they are placed in the unforeseen circumstances of technological/pedagogical teaching context, they might suffer from tension, frustration and fears (Nurit, 2020). For those novices who are less confident, frustration and depression may ultimately drive them from the classroom. In fact, nearly half of all new teachers leave teaching within the first 5 years (Boles, 2002). Therefore, we suggest that future study should include teachers in different stages of career development as the participants to have a more holistic understanding of teachers' agency in emergency online teaching context (Avidov-Ungar et al., 2022).

In addition, we notice that the participants of the study demonstrated different attitudes toward the involvement of parents in emergency online teaching. On the one hand, it proved that the responses of teachers to emergency online teaching were individualized, yet on the other hand, the individualization of the four case study teachers was in such a high degree that it led to the lack of comparability among cases for the author to objectively investigate deeper into the discrepancy in the attitudes of the participants toward parent involvement. Thus, it appears that the study failed to provide an in-depth analysis for this specific issue that readers are interested in learning about. Therefore, we suggest that future study should conduct a more in-depth and thorough investigation with one or two cases on the issue of disparity in teachers' responses to parents' involvement and disruption in teacher-parent relationship in emergency online teaching.

Second, as stated in the article, when teaching was moved from face-to-face offline classroom to virtual online classroom, one of the major challenges that the four case study participants encountered was the ambiguity of their identities and roles (Foreman-Brown et al., 2022). In traditional offline classrooms, most of the teachers regard themselves as knowledge deliverers though sometimes they also think they can be a walking resource center ready to offer whatever help if needed. However, when teaching was moved online, teachers' traditional roles stop working and their sense of professional identity was confronted (Robson, 2017). As a resource, teachers need to change their roles from knowledge deliverers to prompters, in other words, teachers must guide their students to use available resources such as social networking system. On the one hand, teachers might not be as prepared as they should be in terms of digital literacy and online pedagogical repertoire (Noor et al., 2020), and on the other hand, with the traditional lecturing teaching mode students are easily bored and the classroom could easily be out of control. Therefore, the inability to cope with the abrupt shift of teacher identity inevitably has led to tensions such as low agency, greater responsibility, and low professional preparation (Mostafa, 2021). As far as we are concerned, this factor should be included in the future research studies.

Another challenge of emergency online teaching on language teachers manifested by this article is the change of power relationship between teachers and students, and even between teachers and parents. When the habitual teaching approach broke down in emergency online teaching, teachers were forced to reconsider the relationship between students and themselves. According to Ashton (2022), in traditional face-to-face teaching context, teachers' major concern was students' academic needs. Teachers were forced to realized that students' emotional and pastoral needs were more important in emergency online teaching. As a result, teachers had to change the power distance with the students and spent more time in nurturing students' emotional health and well-being. To put it another way, teachers need to become the “leader” and “accompanier” of students through effective guidance and communication (Yao et al., 2020) in emergency online teaching. Meanwhile, the power distance between teachers and parents also changed. While parents only get to go to the school on specific occasions in off-line teaching context, with emergency online teaching, parents were able to be constantly on the background observing the class and monitoring their children, which could result in tension between teachers and parents and led to the self-consciousness of teachers as being “watched” and “on display”. As we see it, future study in teacher agency could track how teachers cope with changes in power relationship with students and parents to better understand the cognitive world of teachers in emergency online teaching.

Third, besides the influencing factors discussed in the article, there is yet another important factor that can influence teacher's enactment of agency in emergency online teaching, that is, the influence of digital affordances on teacher agency in emergency online teaching context (Chen, 2022). As Vongalis-Macrow (2007) points out, digital affordances are another critical factor in realizing the enactment of teacher agency. Studies have shown that despite the limitations on digital affordances for remote teaching, the desire to maintain the pedagogical objectives of face-to-face courses compelled teachers to make the most of their teacher agency by exploring diverse instructional technologies to engage students online (Chen, 2022). As stated in the introduction, there is a distinctive difference between general remote teaching and emergency online teaching. General online teaching, or planned online teaching, differs from emergency online teaching in that online curriculum design and pedagogical decisions are carefully planned and considered beforehand in general online teaching (Hodges et al., 2020). While the digital affordances compelled teachers to realize their role in general online teaching, it largely remains unknown how the limitation of digital affordances influences teachers' enactment of teacher agency in emergency online teaching context. It is pity that this article almost left this factor untold. Hence, we suggest the investigation of interaction between digital affordance and teacher agency should be another direction for future study of teacher agency in emergency online teaching.

## Conclusion

To sum up, we find this article a timely piece, clearly-illustrated and thought provoking. It revealed that language teachers were willing to adapt to their new situated teaching environment by exercising and exerting their agentic actions in emergency online teaching context, that language teachers are social learners and through their personal reflection they would stimulate teacher agency and take actions accordingly, and that they were all able to exercise their teacher agency in line with their new professional identity. It also helps bring to the spotlight problems and challenges arising from emergency online teaching. Through the theme of critical incidents and focus on the practical-evaluative dimension of the cordial triad of agency, the study provides insights and references as to how teachers can enact teacher agency in difficult teaching context. Hence, we have no hesitation in recommending it to anyone who is interested in the interplay of teacher agency and changing instructional environment.

## Author contributions

QC drafted the opinion article. As QC's supervisor, XZ helped her select the commented article, provided insights and suggestions during her writing and did the revision for the text. Both authors contributed to the article and approved the submitted version.

## Conflict of interest

The authors declare that the research was conducted in the absence of any commercial or financial relationships that could be construed as a potential conflict of interest.

## Publisher's note

All claims expressed in this article are solely those of the authors and do not necessarily represent those of their affiliated organizations, or those of the publisher, the editors and the reviewers. Any product that may be evaluated in this article, or claim that may be made by its manufacturer, is not guaranteed or endorsed by the publisher.

## References

[B1] Ashton K. (2022). Language teacher agency in emergency online teaching. System 105, 102713. 10.1016/j.system.2021.10271335059667

[B2] Avidov-Ungar O. Hadad S. Shamir-Inbal T. Blau i. (2022). “professional development processes of teachers in different career stages and in different Covid-19 pandemic periods,” in Proceedings of Society for Information Technology and Teacher Education International Conference, ed E. Langran. (San Diego, CA, United States: Association for the Advancement of Computing in Education (AACE)). 377–383. Available online at: https://www.learntechlib.org/primary/p/220754/https://www.learntechlib.org/primary/p/220754/ (accessed July 25, 2022).

[B3] Biesta G. Priestley M. Robinson S. (2017). Talking about education: exploring the significance of teachers' talk for teacher agency. J.Curric. Stud. 49, 38–54. 10.1080/00220272.2016.1205143

[B4] Boles K. C. Troen V. (2002). Who's Teaching Your Children: Why the Teaching Crisis is Worse Than You Think and What Can Be Done About It. New Haven: Yale University Press.

[B5] Burkhauser M. A. Lesaux N. K.. (2017). Exercising a bounded autonomy: novice and experienced teachers' adaptations to curriculum materials in an age of accountability. J. Curric. Stud. 49, 291–312. 10.1080/00220272.2015.1088065

[B6] Chen M. (2022). Digital affordances and teacher agency in the context of teaching Chinese as a second language during COVID-19. System 105. 10.1016/j.system.2021.102710

[B7] Emirbayer M. Mische A. (1998). ‘What is agency?' Am. J. Sociol. 103, 962–1023. 10.1086/231294

[B8] Foreman-Brown G. Fitzpatrick E. Twyford K. (2022). Reimagining teacher identity in the post-Covid-19 university: becoming digitally savvy, reflective in practice, collaborative, and relational, Educ. Dev. Psychol. 8, 1–9. 10.1080/20590776.2022.2079406

[B9] Gacs A. Goertler S. Spasova S. (2020). Planned online language education versus crisis-prompted online language teaching: lessons for the future. Foreign Lang. Ann. 53, 380–339. 10.1111/flan.12460

[B10] Gao X. Zhang J. (2020). Teacher learning in difficult times: examining foreign language teachers' cognitions about online teaching to tide over COVID-19. Front. Psychol.11, 549653. 10.3389/fpsyg.2020.549653 33071866 PMC7533586

[B11] González-Lloret M. (2020). Collaborative tasks for online language teaching. Foreign Lang. Ann. 53, 260–269. 10.1111/flan.12466

[B12] Hitlin S. Elder G. H. (2007). Time, self, and the curiously abstract concept of agency. Sociol. Theory 25, 170–191. 10.1111/j.1467-9558.2007.00303.x

[B13] Hodges C. Moore S. Lockee B. Trust T. Bond A. (2020). The Difference Between Emergency Remote Teaching and Online Learning, Educause. Available online at: https://er.educause.edu/articles/2020/3/the-difference-between-emergency-remote-teaching-andonline-learning (accessed July 25, 2022).

[B14] Mostafa N. Haniye S. (2021). Covidentity: examining transitions in teacher identity construction from personal to online classes, Euro. J. Teach. Edu. 10.1080/02619768.2021.1920921 (accessed July 25, 2022).

[B15] Noor S. Isa F. Md. Mazhar F. F. (2020). Online teaching practices during the COVID-19 pandemic. Educ. Process: Int. J. 9, 169–184. 10.22521/edupij.2020.93.433845828

[B16] Nurit D. Orna S. (2020). Novice teachers in a changing reality. Europ. J. Teach. Educ. 43, 639–656. 10.1080/02619768.2020.1821360

[B17] Priestley M. Biesta G. Robinson S. (2013). “Teachers as agents of change: teacher agency and emerging models of curriculum”. in Reinventing the curriculum: New trends in curriculum policy and practice, eds M. Priestley and G. J. J. Biesta. (London: Bloomsbury Publishing). 187–206. 10.5040/9781472553195

[B18] Priestley M. Biesta G. Robinson S. (2015b). “Teacher agency: what is it and why does it matter?” in Flip the system: Changing Education from the Ground Up, eds *R. Kneyber and J. Evers*. (Routledge).

[B19] Priestley M. Biesta G. Robinson S. (2015a). Teacher Agency: An Ecological Approach. London: Bloomsbury Publishing. 10.4324/9781315678573-15

[B20] Priestley M. Edwards R. Priestley A. Miller K. (2012). Teacher agency in curriculum making: agents of change and spaces for manoeuvre. Curric. Inq. 42, 191–214. 10.1111/j.1467-873X.2012.00588.x

[B21] Robson J. (2017). Performance, structure and ideal identity: reconceptualising teachers' engagement in online social spaces, Br. J. Educ. Tech. 49, 439–450. 10.1111/bjet.12551

[B22] Ruan X. Zheng X. (2019). The rhetoric and the reality: exploring the dynamics of professional, agency in the identity commitment of a Chinese female teacher. Learn. Cult. Social Interact. 21, 348–361. 10.1016/j.lcsi.2019.04.008

[B23] Tucker O. G. (2020). Supporting the development of agency in music teacher education. J. Music Teach. Educ. 29, 24–36. 10.1177/1057083719885868

[B24] Vongalis-Macrow A. (2007). I, teacher: re-territorialization of teachers' multi-faceted agency in globalized education, Br. J. Sociol. Educ. 28, 425–439. 10.1080/01425690701369376

[B25] Yao J. Rao J. Jiang T. Xiong C. (2020). What role should teachers play in online teaching during the COVID-19 pandemic? Evidence from China. Sci. Insigt. Edu. Front. 5, 517–524. 10.15354/sief.20.ar035

[B26] Zhang C. Yan X. Wang J. (2021). EFL teachers' online assessment practices during the COVID-19 pandemic: changes and mediating factors. Asia-Pacific Edu. Res. 30, 499–507. 10.1007/s40299-021-00589-3

[B27] Zheng X. Borg S. (2014). Task-based learning and teaching in China: secondary school teachers' beliefs and practices. Lang. Teach. Res. 18, 205–221. 10.1177/1362168813505941

